# Supplementary material for: Identification and functional analysis of non-coding regulatory small RNA FenSr3 in Bacillus amyloliquefaciens LPB-18
Source: PeerJ. 2023 May 15;11:e15236. doi: 10.7717/peerj.15236 (PMC10194069; doi:10.7717/peerj.15236)
Supplement: Supplemental Information 4 [file peerj-11-15236-s004.zip › KO/CK-vs-T1_map/map01523.html]

KEGG PATHWAY: Antifolate resistance - Reference pathway


|  |  |
| --- | --- |
| **Antifolate resistance - Reference pathway** |  |

[
Pathway menu
| Organism menu
| Pathway entry
| Show description
| User data mapping
]

Since the 1940s, antifolates have played a pivotal role in drug treatment of malignant, microbial, parasitic and chronic inflammatory diseases. The molecular basis of the anti-proliferative activity of antifolates relies on inhibition of key enzymes in folate metabolism, which results in disruption of purine and thymidylate biosynthesis, inhibition of DNA replication and cell death. The anti-inflammatory properties of antifolate have been most strongly related to its ability to block the release of pro-inflammatory cytokines such as tumour necrosis factor (TNF)-alpha or interleukin (IL)-1beta. Cells may develop resistance to an antifolate drug by virtue of impaired drug transport into cells, augmented drug export, impaired activation of antifolates through polyglutamylation, augmented hydrolysis of antifolate polyglutamates, increased expression and mutation of target enzymes, and the augmentation of cellular tetrahydrofolate-cofactor pools in cells.

|  |  |  |
| --- | --- | --- |
| Reference pathway | 184% 150% 122% 100% 82% 67% 55% | 图片下载 |
